# The Preventive Effect of Dysphagia Screening on Pneumonia in Acute Stroke Patients: A Systematic Review and Meta-Analysis

**DOI:** 10.3390/healthcare9121764

**Published:** 2021-12-20

**Authors:** Seoyon Yang, Yoo Jin Choo, Min Cheol Chang

**Affiliations:** 1 Department of Rehabilitation Medicine, Ewha Woman’s University Seoul Hospital, School of Medicine, Ewha Woman’s University, Seoul 07804, Korea; seoyonyang@gmail.com; 2 Department of Physical Medicine and Rehabilitation, College of Medicine, Yeungnam University, Daegu 41061, Korea

**Keywords:** dysphagia, pneumonia, mortality, screening, prevention, meta-analysis

## Abstract

(1) Background: Dysphagia is common in acute stroke patients and is a major risk factor for aspiration pneumonia. We investigated whether the early detection of dysphagia in stroke patients through screening could prevent the development of pneumonia and reduce mortality; (2) Methods: We searched the PubMed, Embase, Cochrane Library, and Scopus databases for relevant studies published up to November 2021. We included studies that performed dysphagia screening in acute stroke patients and evaluated whether it could prevent pneumonia and reduce mortality rates. The methodological quality of individual studies was evaluated using the Risk Of Bias In Non-randomized Studies of Interventions tool, and publication bias was evaluated by the funnel plot and Egger’s test; (3) Results: Of the 6593 identified studies, six studies met the inclusion criteria for analysis. The screening group had a significantly lower incidence of pneumonia than the nonscreening group did (odds ratio (OR), 0.60; 95% confidence interval (CI), 0.42 to 0.84; *p* = 0.003; I^2^, 66%). There was no significant difference in mortality rate between the two groups (OR, 0.61; 95% CI, 0.33 to 1.13; *p* = 0.11; I^2^, 93%); (4) Conclusions: Early screening for dysphagia in acute stroke patients can prevent the development of pneumonia.

## 1. Introduction

Dysphagia develops in over half of patients with acute stroke [1]. Dysphagia is a major risk factor for aspiration pneumonia in stroke patients [2]. The incidence of aspiration pneumonia in stroke patients with dysphagia is reported to be 20–47% [3,4]. Pneumonia in stroke patients results in prolonged hospitalization and poor clinical outcomes and increases the 30-day mortality threefold [5]. Therefore, the early detection of dysphagia in stroke patients by screening would prevent the occurrence of pneumonia and result in good clinical outcomes.

Previous studies have evaluated the preventive effect of dysphagia screening on the development of aspiration pneumonia in patients with acute stroke [6,7,8,9,10,11]. However, a systematic analysis combining the results of these previous studies has not been conducted. Therefore, to accurately determine the effect of dysphagia screening in acute stroke patients, we conducted a systematic review and meta-analysis of all available and relevant clinical studies related to this topic.

## 2. Methods

### 2.1. Search Strategy

In this study, the PICO (population, intervention, comparison, outcome) model for establishing the search strategy was set as follows: (1) population—acute stroke patients, (2) intervention—dysphagia screening, (3) comparison—no dysphagia screening, and (4) outcome—the occurrence of pneumonia and number of deaths. This meta-analysis was conducted according to the Preferred Reposting Items for Systematic Reviews and Meta-analysis (PRISMA) guidelines [12]. We systematically searched for relevant articles published up to November 2021 in the PubMed, Embase, Cochrane Library, and Scopus databases. The following keywords were used in the search: “dysphagia”, “swallowing”, “deglutition disorders”, “deglutition”, “mass screening”, “early diagnosis”, and “screening”.

### 2.2. Study Selection

We applied the following inclusion criteria for the selection of articles: (1) acute stroke patients were recruited for the study; (2) dysphagia screening was conducted in the intervention group (dysphagia screening group) and not in the control group; and (3) the development of pneumonia or number of deaths was evaluated in both groups. The exclusion criteria were as follows: (1) case reports, reviews, letters, or other undistinctive forms; (2) the same data published repeatedly; or (3) study outcomes not reported.

### 2.3. Data Extraction

All data were independently extracted by two researchers (YJC and MCC) using a standard data collection form. Discrepancies were resolved through discussions with another investigator (SY) and by referring to the original articles. Subsequently, data including the name of the first author, year of publication, sample size, demographic data, dysphagia screening tool, and outcome measures (development of pneumonia and number of deaths) were independently extracted from each eligible article.

### 2.4. Quality Assessment

The methodological qualities of the studies included in the present meta-analysis were evaluated using the Risk of Bias in Nonrandomized Studies of Interventions (ROBINS-I) tool [13]. ROBINS-I was used to determine bias due to confounding, the deviation from intended interventions, missing data, bias in selection of participants into the study, classification of interventions, measurement of outcomes, and selection of reported data in nonrandomized controlled trials. Judgments of bias were expressed as “low risk”, “high risk”, or “unclear risk”.

### 2.5. Statistical Analysis

RevMan software (version 5.3; http://tech.cochrane.org/revman (accessed on 12 November 2021)) was used for statistical analysis of the pooled data. In each analysis, a heterogeneity test was performed using I^2^ statistics, which measures the extent of inconsistency among the results. If I^2^ was ≥50%, the data were considered significantly heterogeneous and a random-effects model was used for data analysis. If I^2^ was <50%, the data were considered homogeneous and a fixed-effects model was applied. We analyzed odds ratios (ORs) to evaluate differences in outcome measures (the development of pneumonia and number of deaths) in the dysphagia screening and control groups. The fixed- and random-effects models were selected according to the different heterogeneity levels of the ratio outcomes. Further, 95% confidence intervals (CIs) were used in the analysis. Statistical significance was set at *p* < 0.05.

### 2.6. Publication Bias

A funnel plot and Egger’s test were used to evaluate publication bias and were analyzed using R version 4.1.2 (R Foundation, Vienna, Austria). A funnel plot was used to visually evaluate whether the individual studies were symmetrical based on the pooled estimate. Egger’s test is a statistical method used to test whether the results of the funnel plot are symmetric. Statistical significance was set at *p* < 0.05.

## 3. Results

### 3.1. Study Selection

**Figure 1:**
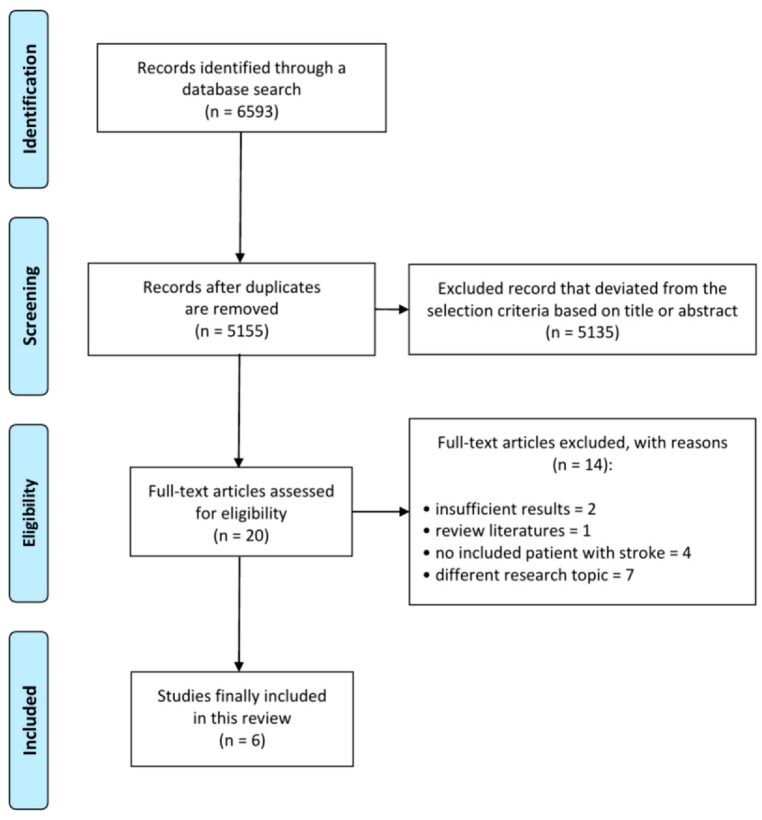
Flow diagram showing the search results of the meta-analysis.

A total of 6593 articles were obtained from the databases, and 1438 duplicated articles were removed (Figure 1). Eligibility screening was conducted by reviewing the title and abstract, and 20 articles were included for full-text reading. After a detailed assessment, 14 articles were excluded: two studies reported insufficient results, one study was a literature review, four studies were not conducted in stroke patients, and seven studies evaluated different research topics. Therefore, a total of six retrospective observational studies [6,7,8,9,10,11] were included to determine the preventive effect of dysphagia screening in patients with acute stroke. The characteristics of the studies included in this analysis are presented in Table 1.

### 3.2. Study Characteristics

All six studies [6,7,8,9,10,11] included in this meta-analysis were retrospective observational studies. In all studies, dysphagia screening was performed as an intervention, and dysphagia screening was not performed in the control group.

Hichey et al. [6] selected patients with acute ischemic stroke from 15 acute care institutions. The dysphagia screen included a water-swallow test, speech therapy, clinical examination, bedside evaluation, modified diet, and nothing by mouth.

Schrock et al. [7] included patients with ischemic and hemorrhagic stroke and performed an emergency department (ED) dysphagia screening. The ED dysphagia screen is a screening method developed by Schrock et al. [14] with the help of neurology and speech pathology for use in all ED patients. The ED dysphagia screen includes questions such as: (1) Is alertness level insufficient to remain awake for 10 min while sitting upright? (2) Is the voice weak, wet, or abnormal in any way? (If cannot speak, circle yes); (3) Does the patient drool? (4) Is the speech slurred? (5) Is the patient’s cough weak or inaudible? (If cannot cough, circle yes).

Sorensen et al. [8] selected acute stroke patients with moderate to severe dysphagia, and Teuschl et al. [9] included patients with acute stroke. Sorensen et al. [8] and Teuschl et al. [9] performed a dysphagia screen using the Gugging Swallowing Screen (GUSS). The GUSS is a dysphagia screening test for stroke patients and consists of an indirect and a direct swallowing test. Indirect swallowing tests confirm swallowing of saliva, level of consciousness, and the ability to cough. In the direct swallowing test, patients try different diets in the order of semi-solid, liquid, and solid, and are assessed for signs of aspiration depending on the type of diet.

Titsworth et al. [10] used a nurse-administered bedside dysphagia screen in patients with ischemic or hemorrhagic stroke. The preprinted stroke order set was modified to include nothing per mouth, including medications as the only diet order. Additionally, a modified nursing dysphagia screen (MNDS) was implemented. The MNDS includes the following questions: (1) Is the patient somnolent (not awake and alert)? (2) Is the patient wet with a gurgly voice on speech or breathing? (3) Does the patient have dysarthria (slurred speech)? (4) Is the patient coughing or choking while breathing or talking? (5) Does the patient have difficulty with oral secretions requiring suctioning? (6) Does the patient/family report that the patient is unable to swallow or has had difficulty swallowing in the past? When patients failed the MNDS, speech-pathology and swallow evaluations were expedited.

Yeh et al. [11] included patients with acute stroke admitted to an intensive care unit and performed dysphagia screening with a 3-step swallowing screen. The first step defined the exclusion criteria for reduced consciousness, dysphagia, need for tube feeding, intubation, lack of oxygen saturation, or frequent choking of saliva. The second step was to swallow 3 mL of water in three trials. The third step was to swallow 100 mL of water twice. The failure criteria for the water-swallow portion of the test included wet voices, slow swallowing, or choking. If a patient failed the water trial, a speech-language pathologist was consulted for a formal evaluation.

### 3.3. Risk of Bias

**Figure 2:**
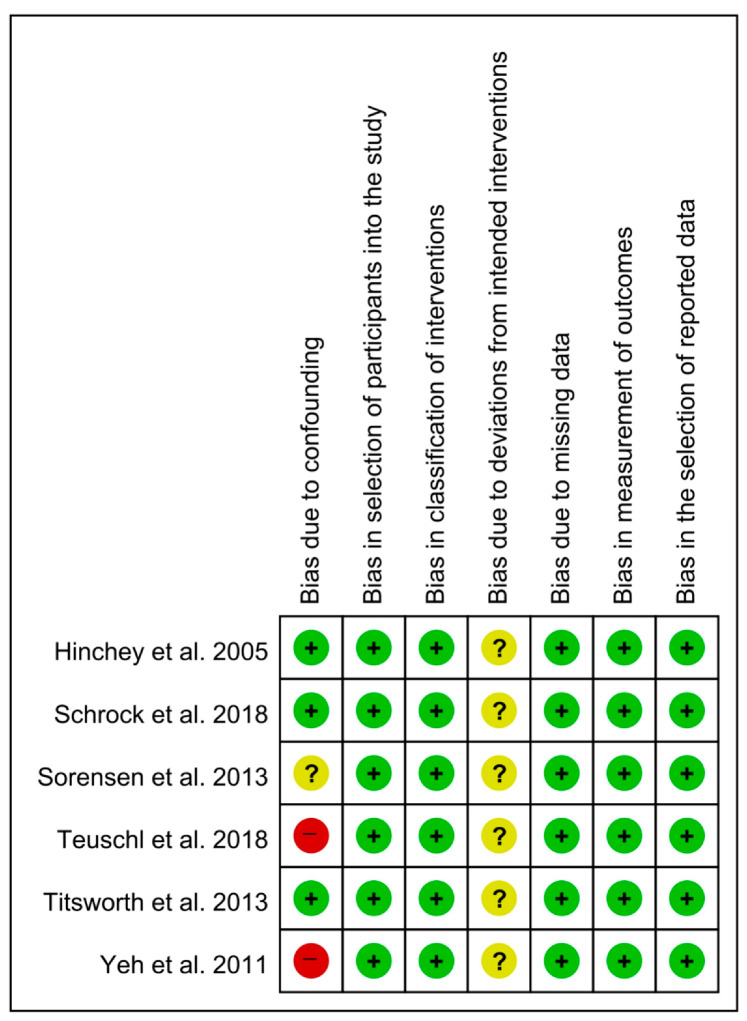
Results of quality assessment of the selected studies [6,7,8,9,10,11].

In the confounding domain, three studies [6,7,10] had a low risk of bias, two studies [9,11] had a high risk of bias, and one study [8] had an unclear risk of bias. All studies [6,7,8,9,10,11] had a low risk of bias in the domains of selection bias, classification of interventions, missing data, measurement of outcomes, and reported results. In the intended intervention domain, all studies [6,7,8,9,10,11] had an unclear risk of bias (Figure 2).

### 3.4. Meta-Analysis Results

**Figure 3:**
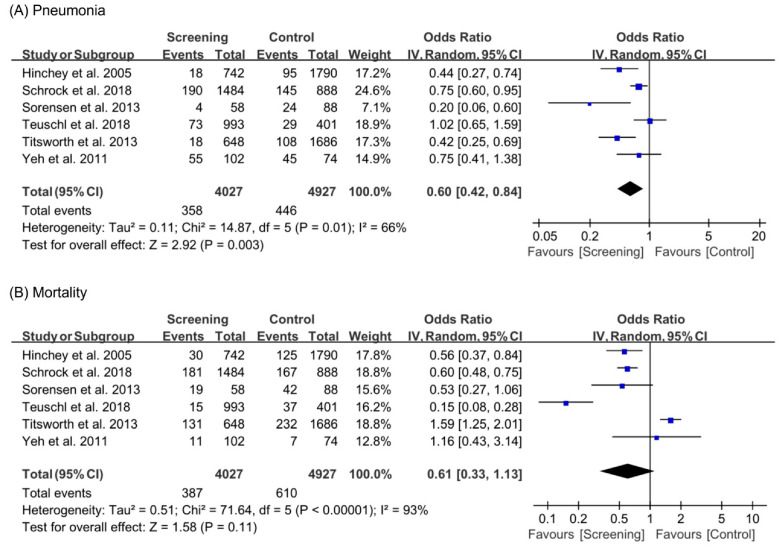
Forest plot showing the results of (**A**) pneumonia and (**B**) mortality after dysphagia screening in acute stroke patients.

In this meta-analysis, the incidence of pneumonia and mortality rates were investigated. A total of six studies [6,7,8,9,10,11] were included in the analysis, including 4027 participants in the screening group and 4927 participants in the control group. Inverse variance was used as the statistical method, and ORs were used to measure the effect size. As I^2^ was >50% in both variables, a random-effects model was adopted. The pneumonia rate was significantly lower in the screening group than in the control group (OR, 0.60; 95% CI, 0.42 to 0.84; *p* = 0.003; I^2^, 66%), but the mortality rate was not significantly different between the two groups (OR, 0.61; 95% CI, 0.33 to 1.13; *p* = 0.11; I^2^, 93%) (Figure 3).

### 3.5. Publication Bias

**Figure 4:**
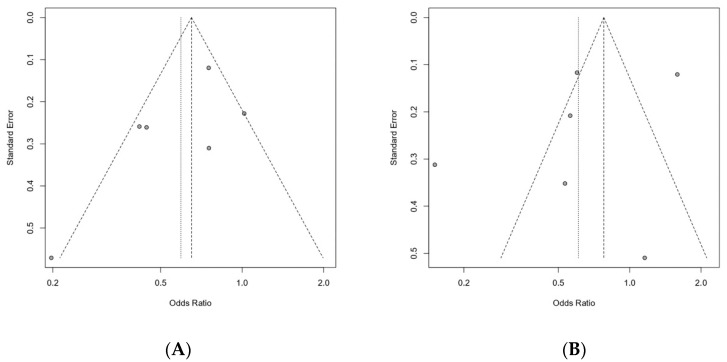
Graphic funnel plot of the included studies. (**A**) Pneumonia, (**B**) Mortality.

Two authors (YJC and MCC) individually assessed the publication bias based on two distinct methods. Publication bias was evaluated using a funnel plot and Egger’s test. On visual examination, the funnel plot was considered symmetrical for pneumonia but asymmetric for mortality. However, the results of Egger’s test were not significant, indicating no publication bias (pneumonia, *p* = 0.3607; mortality, *p* = 0.4333) (Figure 4).

## 4. Discussion

In our meta-analysis, we found that dysphagia screening can prevent the development of pneumonia in patients with acute stroke.

Various tools have been used in previous studies for dysphagia screening. A swallowing test with water or a semisolid food and a survey asking about the presence of aspiration and swallowing function were used to evaluate the presence of dysphagia [15,16,17,18]. The videofluoroscopic swallowing study (VFSS) is a gold-standard tool for assessing swallowing disorders and oropharyngeal aspiration [19]. However, a fluoroscopic machine is necessary for a VFSS. Clinicians should schedule the use of fluoroscopic machines and prepare materials for conducting a VFSS, such as contrast or foods or materials mixed with contrast [20]. Compared with VFSS, dysphagia screening is relatively convenient. Although the diagnostic accuracies of the dysphagia screening tools used in the previous studies would be not as accurate as that of VFSS, they resulted in a reduction in the occurrence of pneumonia in acute stroke patients [6,7,8,10,11]. Therefore, dysphagia screening can be useful in clinical practice, and to prevent pneumonia, we think it should be recommended for stroke units. In patients who showed dysphagia during dysphagia screening, active rehabilitation for treating dysphagia and nonoral feeding methods should be applied.

In our study, dysphagia screening did not reduce the mortality rate after stroke, although this result is contradictory to previous reports [21,22]. Despite the reduction in the development of pneumonia, the mortality rate was not significantly reduced in patients who underwent dysphagia screening. We believe that many other factors, such as severe brain damage and poor medical conditions induced by disorders other than pneumonia, could have been involved in patient deaths. These may have been confounding factors in the previous studies. In addition, the number of subjects included in the previous studies might not be enough to present a significantly different mortality rate between the dysphagia screening and control groups. In addition, all the studies included in our meta-analysis were retrospective observational studies. Considering the characteristics of the intervention, a randomized controlled prospective trial was not possible, due to practical and ethical reasons. As the included studies were conducted retrospectively, the allocation of patients in the intervention and control groups was not consistent across studies. In the study by Sorensen et al. [8], the patients in the intervention group were prospectively recruited (from 2009 and 2010) and were compared with the internal historic control group with different time periods (retrospectively selected from 2008 and 2009). Such a design may have introduced selection bias. Another limitation of this meta-analysis was that the screening methods differed across the included studies. Some studies included interventions such as diet modification or speech therapy, in addition to dysphagia screening. Including other methods of intervention may have influenced the overall rate of pneumonia and mortality.

In conclusion, we found that dysphagia screening is effective in preventing the development of pneumonia in patients with acute stroke. We believe that dysphagia screening in stroke patients can be helpful in improving functional outcomes and preventing the occurrence of pneumonia. As randomized controlled trials (RCTs) were not included in our review, further well-designed prospective RCTs are necessary to draw further definite conclusions on this topic. Additionally, studies investigating the most appropriate dysphagia screening tool should be conducted in the future.

## Figures and Tables

**Table 1:** Characteristics of included studies.

Outcomes	Screen Methods	Definition of Pneumonia				Intervention/Control Group	Age (I/C, y)		Screening (I)/no Screening (C) (n)	Study Design	Study	No.
Pneumonia rate, adherence rate, mortality, stroke severity (National Institutes of Health Stroke Scale), length of stay in hospital	Water-swallow test, speech therapy, clinical examination, bedside evaluation, modified diet, and nothing by mouth	The definition of pneumonia includes either the clinical finding of rales or dullness to percussion and 1 of the following: purulent sputum, or isolation of the organism, or chest radiograph showing evidence of an infiltrate/consolidation/cavitation or pleural effusion and 1 of the following: purulent sputum or isolation of the agent or antibody evidence of an agent.				A formal dysphagia screen vs. no formal screen	Mean age (SD): 71.3 ± 14/68.7 ± 15		18/95	RO	Hinchey et al. 2005 [6]	1
Pneumonia rate, mortality, stroke severity (National Institutes of Health Stroke Scale), subarachnoid hemorrhage severity (Hunt-Hess Score)	Emergency department dysphagia screen	Pneumonia was pre-defined as a new infiltrate on chest radiogram that was treated with antibiotics.				Before vs. after use of dysphagia screen	Patients with acute ischemic stroke cohort; median age (IQR): 63 (53–76)/64 (56–76)	Patients with intracranial hemorrhage; median age (IQR): 61 (50–70)/64 (54–77)	190/145	RO	Schrock et al. 2018 [7]	2
Pneumonia rate, mortality, stroke severity (Scandinavian Stroke Scale score), functional status (Barthel-100 score), length of stay in hospital	GUSS	Pneumonia was categorized into two categories:	1. “Possible pneumonia” if C-reactive protein >50 mg/L and/or leukocyte count >10 × 10^9^/L and accompanied by respiratory symptoms such as coughing (with or without expectoration), dyspnea, tachypnea >20/min, and/or O_2_ saturation <90%. All but one of the patients in the intervention and the internal control groups had a chest X-ray performed to verify the pneumonia.	2. “X-ray verified pneumonia” if infiltrative changes were observed by chest X-ray, which could be explained by pneumonia, accompanied with C-reactive protein >50 mg/L and/or leukocyte count >10 × 10^9^/L and/or respiratory symptoms.	The incidence of the clinical variables described above was recorded within ±3 days of the qualifying pneumonia.	GUSS method for dysphagia screening vs. control group selected retrospectively at two consecutive time points	Median age (IQR): 85 (78–89)/84 (79–88)		4/24	RO	Sorensen et al. 2013 [8]	3
Pneumonia rate, mortality, stroke severity (National Institutes of Health Stroke Scale), functional status (modified Rankin scale, Barthel index), complications	GUSS	Diagnosis criteria for pneumonia were based on the modified CDC criteria and the recommendations from the pneumonia in the stroke consensus group for probable SAP: clinical symptoms (e.g., cough, purulent sputum) in combination with clinical signs such as fever, rales, bronchial breath sounds, or elevation of inflammatory markers in laboratory tests confirmed by at least one chest X-ray within 7 days after stroke. Pneumonia diagnosed later than 7 days after admission was defined as hospital-associated pneumonia.				Screening vs. no screening	Median age (IQR): 70 (59–82)/77 (67–84)		73/29	RO	Teuschl et al. 2018 [9]	4
Pneumonia rate, mortality, stroke severity (National Institutes of Health Stroke Scale)	Nurse-administered bedside dysphagia screen	The CDC and National Health Safety Network criteria for clinically defined pneumonia were used for HAP. In brief, the subject had to have ≥2 serial radiographs with 1 of the following: a new infiltrate, consolidation, or cavitation. Second, the patient had to have 1 of the following: fever >38 °C, leukopenia or leukocytosis, or altered mental status. Finally, they had to have 2 of the following: new onset of purulent sputum, new onset of worsening cough, dyspnea, or tachypnea, rales or bronchial breath sounds, or worsening gas exchange by oxygen saturation or arterial blood gas.				Screening vs. no screening	Mean age (SD): 63.8 ± 15.4/63.6 ± 16.1		18/108	RO	Titsworth et al. 2013 [10]	5
Pneumonia rate, mortality, stroke severity (National Institutes of Health Stroke Scale), length of stay in hospital	Three-Step Swallowing Screen protocol	The diagnosis of pneumonia was based on the CDC definition of nosocomial pneumonia as follows: (1) rales in breathing sound examination or dullness in chest percussion, or (2) radiological evidence of new infiltration, consolidation, cavitation, or pleural effusion, and with at least one of the following findings: (a) new onset of purulent sputum, (b) positive blood culture, and (c) positive sputum culture.				Prescreening group vs. postscreening group	Mean age (SD): 64.4 ± 13.3/69.9 ± 13.7		55/45	RO	Yeh et al. 2011 [11]	6

C, control group; CDC, Centers for Disease Control; GUSS, Gugging Swallowing Screen; HAP, hospital-acquired pneumonia; I, intervention group; IQR, interquartile range; RO, retrospective observational; SAP, stroke-associated pneumonia; SD, standard deviation; y, years.

## Data Availability

Not applicable.
